# Acute Pancreatitis during and after Pregnancy: A Review

**DOI:** 10.3390/jcm13072028

**Published:** 2024-03-30

**Authors:** Alberto Maringhini, Margherita Rossi, Rosalia Patti, Marco Maringhini, Valerio Vassallo

**Affiliations:** 1 Internal Medicine, Azienda di Rilievo Nazionale e di Alta Specializzazione (ARNAS) Civico, 90127 Palermo, Italy; margherita.rossi@arnascivico.it (M.R.); marco.maringhini@arnascivico.it (M.M.); valerio.vassallo@arnascivico.it (V.V.); 2 Pancreas Unit, Azienda di Rilievo Nazionale e di Alta Specializzazione (ARNAS) Civico, 90127 Palermo, Italy; rosalia.patti@arnascivico.it

**Keywords:** pancreatitis, pregnancy, gallstones, biliary sludge, hypertriglyceridemia

## Abstract

During pregnancy and in the post-partum period, several diseases may arise or become exacerbated. Acute pancreatitis is an inflammatory disease with an increasing incidence in Western countries. The incidence of acute pancreatitis during pregnancy is not different with respect to the general population, but this incidence increases in the first 2 years after delivery. Biliary sludge and stones are the most frequent aetiologies, followed by hypertriglyceridemia. Taking care of the mother and foetus through a potentially severe disease requires a team consisting of an obstetrician, a gastroenterologist, an anaesthesiologist, and a surgeon. It is necessary to monitor the health of the foetus/child and the mother during pregnancy, childbirth, and puerperium. The management of this care depends on the systemic and local complications, the severity of the acute pancreatitis, and the trimester of pregnancy. Some diagnostic tools and many drugs are not safe for foetuses, while interventional endoscopy and surgery have limitations and can only be used after an accurate evaluation of benefit/risk ratios. Despite these limitations, maternal mortality due to acute pancreatitis is low during pregnancy, mainly thanks to multidisciplinary approaches for these patients. A careful diet to prevent obesity, alcohol abstinence, routine serum triglyceride control, and breastfeeding for at least three months may prevent acute pancreatitis during and after pregnancy.

## 1. Introduction

During pregnancy and in the post-partum period, several diseases may arise or become exacerbated. Some diseases are typically and exclusively related to pregnancy, others are not specific to this condition but may have a different clinical presentation and/or a peculiar natural history: these diseases may be cared for with a different approach with respect to the general population.

Acute pancreatitis (AP) is a relatively common disease of the exocrine pancreas associated with severe abdominal pain, several organ failures, potential pancreatic necrosis, and a mortality of 1–5%. Overall, it has an average incidence of 30–40 cases/100,000 population per year. The global incidence of AP seems to be increasing in Western countries and its average cost is USD 10,000 per patient [1,2,3,4,5].

Several papers in recent years have changed our understanding of epidemiology as well as our approach to the clinical diagnosis and therapy of AP that take place during or after pregnancy. Therefore, we have checked all papers from 2011 through September 2023 in PubMed, Scopus, and Cochrane research to write this review. We excluded case reports and animal studies from our research. The aim of this review is to highlight what is new in the epidemiological, diagnostic, and therapeutic approaches to AP during and after pregnancy. We accurately selected papers with diagnostic and therapeutic items, highlighting evidence-based procedures and therapies demonstrated by randomized controlled trials.

## 2. Epidemiology

In 2008, a 10-year study of 15 Midwest Hospitals in the United States evaluated 101 cases of AP that occurred during pregnancy among 305,101 deliveries (incidence: 33/100,000 deliveries) [6]; in 2009, a 7-year study in Texas observed 96 cases of AP with a cumulative incidence of 113/100,000 deliveries [7]; in a 5-year single academic centre study in New Jersey (USA, 2013), AP occurred in 29 cases among 25,600 total hospital deliveries (incidence: 99/100,000 deliveries) [8]. All of these three studies were retrospective in tertiary hospitals with selection bias and likely higher incidences with respect to the general population. A cohort population study carried out in California in 2015 [9] registered an incidence of AP in 17/100,000 deliveries. In a recent Sicilian population-based cohort study, AP incidence during pregnancy was not different with respect to the incidence in the general population but it increased in the first 2 years after delivery [10].

The increased incidence after delivery was also evident in a population-based case–control study we conducted at Mayo Clinic (Olmstead County, Minnesota) at the beginning of the 21st century [11]. In this study, we excluded a relationship between pregnancy and AP, but we demonstrated an increased prevalence of gallstones AP in young new mothers in the post-partum period (below 30 years old) [11].

In the Sicilian population study, AP mortality was absent during pregnancy (*n* = 34), while the mortality in the post-partum period (1/287) was lower with respect to the general population’s AP mortality rate (29/1564) [10].

In our Sicilian population-based cohort study, the AP incidence decreased from the first to the last trimester (28.3, 17.7, and 14.1/100,000 women per year); this difference was not significant, but the number of AP cases was low (*n* = 34). This result is in contrast to a recent meta-analysis in which the incidences were, respectively, 3.4, 23.2, and 63.2/100,000 women per year [12].

In 2022, a meta-analysis of 154 papers published by Chinese researchers evaluated a total of 4034 patients with AP admitted to Chinese hospitals during pregnancy: the maternal mortality rate was 2.8% (95% CI 1.5–5.1). In the same study, only 696 patients were stratified for trimester mortality: maternal death rates were 12.7% in the first trimester, 7.9 in the second, and 6.4 in the last [12].

## 3. Aetiologies

### 3.1. Biliary Sludge and Stones

AP may arise in pregnancy, and both statuses are linked to the presence of biliary stones and sludge. The impressing elevated levels of oestrogen and progesterone during pregnancy increase bile cholesterol saturation and lithogenicity and determine bile stasis in the gallbladder [13,14,15,16], which can explain the higher incidence of biliary sludge and stones during the last 6 months of pregnancy: respectively, 31% and 2% [17,18]. In this prospective cohort study, we found that obesity was a predictor of new sludge, and biliary sludge was a risk factor for the development of new stones; in the first year after delivery, sludge and stones spontaneously disappeared in 61% and 28% of cases, respectively [17,19,20]. The predictors of the spontaneous disappearance of stones were a small diameter (<1.0 cm) and an older age of new mothers (>30 years old). Oestrogen and progesterone decreased after delivery, reducing biliary lithogenicity and promoting gallbladder motility. This phenomenon may help with the spontaneous disappearance of most sludge and prime some small stones for dissolution or ejection through the common bile duct [17,19,20].

In a recent Sicilian population cohort study [10] on childbearing-age women recruited in 6 consecutive years (more than 7 million person-years), we demonstrated that AP incidence did not increase during pregnancy (20.02 versus 21.61/100,000 of controls), and its incidence seemed to decline from the first to the last trimester (28.3, 17.7, and 14.1/100,000 women per year); this difference was not significant, but the number of AP cases was low (*n* = 34). This result is in contrast with a recent meta-analysis in which the incidences were, respectively, 3.4, 23.2, and 63.2/100,000 women per year [12]. Incidence of AP cases increased after delivery with a peak in the first semester (95.37/100,000 women per year) with a decline to normal incidence after 2 years. The incidence of gallstones AP post-partum decreased with age, while it increased in non-pregnancy-related GS AP [5,10]. Considering that biliary sludge and stones are more prevalent before delivery [17], we can speculate that gallbladder motility has a significative role in the pathogenesis of gallstones AP. Parity is only a risk factor for gallstones in pregnant young women (<30 years old) [21,22,23]; small stones spontaneously disappear post-partum, mainly in older new mothers (>30 years old) [17,18,19,20]; and AP is significantly more frequent in younger ages [10,11]: all these epidemiological data support an age-related hormonal difference in the post-partum period like the well-known amenorrhea period and anovulatory cycles present in the post-partum period of the oldest new mothers [24]. Missing ovulatory cycles in the first months after delivery may determine lower levels of oestrogen, lower levels of bile cholesterol, and more spontaneous dissolutions of sludge and small stones.

The only physiological event that increases anovulatory cycles and determines longer amenorrhea like advanced age is breastfeeding [5,20,25]; therefore, in a population-based case–control study, we found an impressive protective effect of breastfeeding with a significatively lower prevalence of AP in women having performed at least 3 months of breastfeeding [5,20,25].

### 3.2. Alcohol

Alcohol use is associated with pancreatitis in a dose-dependent relationship [26]. Alcohol consumption may reduce gallstone incidence [27]; however, during pregnancy, it can cause birth defects, growth restrictions, abortions, and foetal alcohol syndrome [28]. So far, no level of alcohol has been demonstrated to be safe during pregnancy, so we discourage any alcohol use during all stages of pregnancy. Prevalence data in the literature are missing in population studies; different values are reported in other studies, from 0 in a retrospective series from China [29] to 12.5% in the 15 Midwest Hospital study in the United States [6]. Of course, tertiary institution data (from single or multiple tertiary institutions) have selection bias and may be not representative of the general population.

### 3.3. Hypertriglyceridemia

Hypertriglyceridemia is a known aetiology of AP [3]. The increased risk of AP occurs when serum triglyceride is above 500 mg/dL, but it is most frequent with triglyceride levels above 1000 mg/dL [3]. Hypertriglyceridemia is related to 4–9% of all AP cases in the general population [30]. The Endocrine Society classifies hypertriglyceridemia as either primary (genetic) or secondary (e.g., diabetes mellitus, metabolic syndrome, alcohol, or pregnancy) [31]. There is a 4% increase in AP incidence for every 100 mg/dL rise in triglyceride levels above 1000 mg/dL [32]. During pregnancy, changes in lipid and carbohydrate metabolism are present to ensure their better availability to the foetus: increased glucose synthesis, progesterone production, lipogenesis, and reduced lipolysis. In women with an altered lipoprotein metabolism, these adaptive changes cause severe hypertriglyceridemia.

In the third trimester of pregnancy, the levels of triglyceride are about 2–4 times the normal value but usually are less than 300 mg/dL. In the Chinese meta-analysis, hypertriglyceridemia was the cause of AP during pregnancy in 35.1% of cases, while a biliary aetiology was the cause in 42.4%, and idiopathic in 16.8% [12].

Serum triglyceride levels should be routinely measured, whether or not there is a familial history of hypertriglyceridemia. The dosage of serum triglyceride must be routinely carried out, including in women without a familial history of hypertriglyceridemia.

The mechanism of hypertriglyceridemia-related AP seems to be excessive local free fatty acids and lysolecithin due to the lipoprotein substrates in pancreatic cells, which modify the acinar cells and the microvascular membranes because albumin exceeds the carrying capacity of the pancreas [33,34].

Hypertriglyceridemia-related AP is associated with the worst prognosis in pregnancy and in the general population [3,32].

### 3.4. Other Aetiologies

Other aetiologies, including drugs, post-ERCP, trauma, genetic, chronic pancreatitis, etc., are extremely rare and their clinical presentation and natural history do not seem to be different with respect to the general population.

## 4. Clinical Presentation and Diagnosis

The presentation of AP includes epigastric or abdominal pain with back irradiation (80–95%), nausea and vomiting (40–80%), fever, abdominal distension, irritability, breathlessness, impaired consciousness, low oxygen saturation, tachypnoea, hypotension, ileus, and/or oliguria [3]. In pregnancy, physical examination is difficult and should be performed with care; the most frequent findings are rebound pain, tenderness, and ileus with no peristalsis [35].

A diagnosis of AP requires two out of three criteria: abdominal pain consistent with AP, serum amylase or lipase levels three or more times the upper normal limit, and cross-sectional imaging consistent with AP (magnetic resonance imaging, MRI, and ultrasounds, USs, in pregnancy, and computed tomography, CT, in non-pregnancy [36].

The diagnosis must be rendered upon admission to the hospital and within 2 days from the beginning of symptoms because usually, the amylase increase returns to normal in 3 days [35]. The first two criteria alone may fail to identify 25% of patients with AP while making false positives in 10% of patients without AP [37].

Other laboratory tests are blood count, electrolytes, urea, creatinine, glucose, C-reactive protein, bilirubin, aspartate and alanine transferase, gamma-glutamyl transpeptidase, triglycerides, and cholesterol level tests. With severe hypertriglyceridemia, serum amylase and lipase may be falsely normal in AP [3]. Initial diagnostic imaging is based on abdominal ultrasounds. During the first days of AP, bowel artefacts reduce the diagnostic accuracy of ultrasonography, but it is useful enough for the diagnosis of biliary sludge and stones, dilated biliary ducts, and the presence of abdominal or pleural effusion; the latter seems to be an accurate predictor of severe disease [38]. When an ultrasound proves completely inadequate, you can use MRI without a contrast agent for a definitive diagnosis of AP and the presence of stones in the gallbladder or biliary duct.

Ultrasounds are considered safe during all stages of pregnancy. The ICNIRP (International Commission on Non-Ionizing Radiation Protection) recommends postponing non-emergency MRI in the last two trimesters because the risks of heating from radiofrequency pulses and acoustic noise on the foetus have not been clarified [39].

According to the 2007 ACR (American College of Radiology) guidelines, MRI can be used in pregnancy at any gestational age if the benefit outweighs the risks, as established by an MRI radiologist. In acute situations, if the benefits outweigh the risks, then an MRI, even in the first trimester, can be carried out. According to the ACR white paper on MRI safety, you must answer the following questions: could you reach a diagnosis using ultrasound? Might MRI change the therapy of the patient? Could this study be postponed until after pregnancy? [39].

Abdominal ultrasound has a sensitivity of 73% and a specificity of 91% in diagnosing common bile duct stones. This accuracy is lower during P for gas artefacts, and it depends on the operator’s experience [39]. When you suspect a common bile duct stone, MRI and endoscopic ultrasound may be necessary. Endoscopic ultrasounds (EUS) are safe during pregnancy but require anaesthetic assistance and must be proposed in referral centres when ERCP and endoscopic sphincterotomy are necessary [39].

CT scans are not useful due to radiation problems.

After a diagnosis of AP is made, the most relevant step is to predict the severity. Predictions of severity are made as early as possible after admission to determine which patients may develop local and/or systemic complications, and who will need intensive management.

According to the prognostic Atalanta criteria, AP severity has been divided into three categories: mild, referring to AP without organ dysfunction or systemic complications; moderately severe, referring to AP with persistent organ dysfunction or localized/generalized complications within 48 h of admission; and severe pancreatitis referring to AP with persistent organ dysfunction or localized/generalized complications for more than 48 h after admission. Organ dysfunction is referred to using the modified Marshall scoring system (Table 1) [36]. Treatment modalities depend on the predicted severity of AP. Many scoring systems using clinical and laboratory or imaging features have been studied for severity prediction, as in the original Atalanta Classification [36]. More recent studies have grouped the moderately severe category (RAC, Revised Atalanta Classification) for a binary prediction of severity [36]. Shown in Table 1, Table 2 and Table 3 are the most common scoring systems used for the prediction of severity [40].

Prognostic factors of severe disease must be carried out in the first 24–48 h.

AP has been divided into three prognostic categories: About 80% of AP cases are mild; patients may be cared for in general hospitals and need enteral or parenteral nutrition, pain drugs, sometimes pump inhibitors for upper GI problems, and antibiotics when cholangitis or other infections are suspected. On the other hand, the remaining 20% of patients have moderate–severe diseases and they need dedicated intensive units to care for complications.

Many efforts have been made by researchers to predict the severity of AP in pregnancy and, recently, several studies have investigated specific predictors of severity for mothers and foetuses [12,40,41,42,43].

The same predictors used in the general population should be used in pregnancy. The choice of scale depends on the experience of the centre. Furthermore, several single predictors have been suggested, like LDH, PCR, Ht, interleukin-6, BMI, pleural effusion, and triglyceride levels [3,35,38].

Several studies beyond this have been published on predictors of the severity of AP in pregnancy, but they are all retrospective, with missing data, and selection bias. We need prospective studies before accepting the routine clinical use of new predictors of severity specific to AP in pregnancy.

## 5. Therapy

Taking care of the mother and foetus through a potentially severe disease requires a multidisciplinary team (an obstetrician, a gastroenterologist, an anaesthesiologist, and a surgeon) monitoring the health of the foetus/child and mother during pregnancy, childbirth, and puerperium.

Any drug administration must be chosen according to categories that guide evidence-based safety as shown in Table 4. Drugs in less safe categories (C, D) may be used if the benefit/risks ratio is adequate. Drugs in category X cannot be used. More recently, the FDA published the Content and Format of Labeling for Human Prescription Drug and Biological Products; Requirements for Pregnancy and Lactation Labeling, for an easy consultation on all drugs you need in pregnancy or lactation [44].

### 5.1. Oxygen

An oxygen saturation (SpO_2_) of 94–98% is a valid target range to prescribe or to be given by an expert with recording of inspired oxygen, the oxygen delivery system, oxygen flow rate, and oxygen saturation, all linked to a track-and-trigger early warning system [45].

### 5.2. Intravenous Fluid Resuscitation

The immediate administration of intravenous fluid is critical in AP, as this compensates for third-space volume loss and tissue hypoperfusion, correcting pancreatic and systemic microcirculatory failures as a consequence of many inflammatory cascades [46]. An early intravenous fluid infusion within 24 h of hospital admission results in lower rates of persistent systemic inflammatory response syndrome and organ failure, suggested to be given at 5–10 mL/kg/h [47]. Fluid resuscitation must decrease and/or patients must maintain a heart rate of <120/min and a urine output of >0.5 mL/kg/h and if non-invasive continuous arterial pressure measurement is present, patients must maintain a mean arterial pressure of 65–85 mm Hg, with haematocrit at 35–44%. Fluid overload is associated with increased evidence of fluid retention without improvement in clinical outcomes [48]. A small trial including 40 non-pregnant patients suggested Ringer’s lactate solution is preferred to saline in the resuscitation phase of pancreatitis [49] but larger trials are needed to have a definitive evidence-based approach to what infusion is better; regardless, at present, Ringer’s lactate is the most used solution for fluid resuscitation in acute pancreatitis [3].

### 5.3. Pain Management

The administration of antispasmodics (drotaverine, hyoscine, and butyl bromide) and analgesics (paracetamol) are permitted. Nonsteroidal anti-inflammatory drugs in the first and third trimesters of P are not indicated; metamizole and opioids are not permitted throughout any stage of pregnancy, but meperidine has been frequently used in pregnancy and labour without any apparent harmful effects. Acetylsalicylic acid, at a low dosage (0.100 mg) is safe [35].

Well-tolerated early oral nutrition, in less severe cases, may reduce pain intensity, duration, and analgesic use, [50]; in severe cases, the recurrence or exacerbation of pain with eating delays the resumption of solid food intake [50].

### 5.4. Nutrition

Moderate and severe AP induces a hypermetabolic state, with lipolysis, protein catabolism, insulin resistance, and loss of body mass, all exacerbated by inadequate nutrition and infection [51,52]. Oral refeeding should be started as soon as tolerated, and if not, liquid food or enteral tube feeding must be given within a day or two of admission [53]. The nasogastric route is easier than the naso-jejunal route, but some patients do not tolerate the former for delayed gastric emptying. Oral or enteral nutrition shows fewer pro-inflammatory responses and reduces bacterial translocation across the gastrointestinal permeability barrier with respect to parenteral nutrition, with risks of catheter placement and infection [54,55]. Enteral tube feeding cannot be considered for patients who are hemodynamically unstable or who are not volume-replete due to the risk of gut injury through non-occlusive mesenteric ischemia [54,55].

### 5.5. Antibiotics

During pregnancy, you have to be careful about antibiotics being transplacentally transferred to the foetus with a risk of teratogenicity.

Patients with mild AP and no cholangitis or respiratory signs suspect of infection do not need antibiotics. When an infection is suspected, the choice of antibiotics is based on the knowledge of their effect on the foetus.

Metronidazole passes across the placenta, but some studies failed to show any association with a teratogenic effect [56]. There are not enough human studies on using Imipenem, which belongs to the carbapenem class of antibiotics [57]. Quinolones have shown some adverse effects in animal studies; however, there are no adequate human studies.

Ampicillin–sulbactam and Piperacillin–Tazobactam can be used. Of course, if the initial antibiotics are ineffective, the therapy will be modified according to cultures and antibiograms. The final decision in an infected patient must be taken looking for the benefits that may outweigh the risks (Table 5) [57].

### 5.6. Management of Underlying Causes

#### 5.6.1. Biliary Stones

AP associated with biliary stones is treated according to previous suggestions, severity, and using drugs that are safe in pregnancy.

A specific issue is the endoscopic treatment of the common bile duct during pregnancy for the prevention of AP relapse or other complications like cholangitis, cholestasis, jaundice, and colic pain.

Another specific issue is surgery during pregnancy for complicated AP or cholecystectomy in the prevention of AP relapse.

#### 5.6.2. ERCP (Endoscopic Retrograde Cholangiopancreatography)

The American Society of Gastrointestinal Endoscopy has published guidelines for endoscopy in pregnancy [58]. Table 6 shows the general principles guiding endoscopy in P [57].

For narcotic analgesics, we remember, in the light of two large studies, [59] that meperidine (category B) does not seem to be teratogenic, and it is preferred to morphine (category C), which crosses the foetal blood–brain barrier faster. Naloxone (category B) crosses the placenta within 2 min of intravenous administration; it does not seem to be teratogenic. It cannot be used in mothers dependent on opiates because of withdrawal symptoms. It must only be used in respiratory depression or hypotension. Benzodiazepines (category D) and flumazenil (category C) cannot be used. Propofol (category B) can only be used by experienced anaesthesiologists. Simethicone (category C) has limited data on pregnancy, but it is usually used.

Glucagon, an antispasmodic agent usually used during ERCP, can be used during pregnancy. Topical anaesthetics such as lidocaine (category B) are not contraindicated when they are necessary (Table 7).

Endoscopy should be deferred until after the first trimester whenever possible, and it needs to have a strong indication with an accurate assessment of risks versus benefits.

ERCP should be used during pregnancy only when therapeutic interventions are needed. Biliary pancreatitis, symptomatic choledocholithiasis, and cholangitis are common indications and can lead to foetal loss. Only expert endoscopists should attempt the procedures. Many studies have confirmed the safety of ERCP in pregnancy [60,61], despite an increased risk of post-ERCP AP (16% in 65 patients) [61]. A major concern is foetal health. The foetus’s radiation exposure depends on many factors, like patient position and protection. To reduce radiation-associated risk, it is suggested to reduce fluoroscopic time and over-radiation time by collimating the radiation beam only to the area of interest. You must avoid taking hard-copy X-ray films. With care, foetal exposure to radiation can be below 50 to 100 mSv, levels considered to be of concern for teratogenesis [62].

You can perform ERCP without fluoroscopy using a wire-guided cannulation technique. EUS may be performed before ERCP for the diagnosis of the number and size of stones; it may also be used later for confirming stone removal.

#### 5.6.3. Surgery

Surgical intervention during pregnancy has some limitations. The first is related to the adverse events of general anaesthesia. The effects of general anaesthesia on the foetus are unknown, and anaesthetics appear to cross the placenta. Any kind of surgery in pregnancy is difficult for physiological and anatomical changes. The main questions for cholecystectomy are: Is it possible to postpone the surgery until post-partum? When is the surgery? What form of surgery is it (open or laparoscopic)?

A cholecystectomy should be executed to prevent AP relapse or other biliary complications. It is well known that after cholecystectomy, the incidence of AP is much lower [63]. In a study combining data from the Connecticut Laparoscopic Cholecystectomy Registry and data from the Connecticut Hospital Association (CHA), all cholecystectomies performed during pregnancies from 1992 through 1996 were evaluated. Clinical data were obtained from 46 patients (20 laparoscopic and 26 open cases). Their conclusions were that laparoscopic cholecystectomy is not associated with increased numbers of foetal complications. Premature uterine contractions are more frequent after open surgery [64]. In a recent systematic review of 590 patients, authors demonstrated that most procedures were performed after the first trimester (70.7%). Intra- and postoperative complications were present in 3.5% and 4% of the study population. The conversion rate to open surgery was 2.2%. Foetal loss and pre-term delivery rates were 0.4% and 5.7% [65].

During pregnancy, biliary AP, with stones smaller than 10 mm in diameter, may be treated with ursodeoxycholic acid after the first trimester, with no teratogenic risk and good results in the dissolution of small stones.

#### 5.6.4. Hypertriglyceridemia

A woman with AP secondary to hypertriglyceridemia must be treated with general suggestions. During the last trimester, the increase in triglyceridemia may be elevated and difficult to control. These patients must receive fluid and glucose with no more than 400 Kcal/day. The oral alimentation must be restarted (as soon as possible) with a diet at 400 Kcal until you obtain a serum level of less than 500 mg/dL. If a low-calorie diet is not enough, the use of low-weight heparin or insulin in low doses may help in lowering chylomicrons.

In a severe case, we obtained a triglyceride control only with plasmapheresis, repeated every two days six times [33,34].

Omega-3 fatty acids may be safely added to an isocaloric diet during AP when oral alimentation is started to keep lower triglyceride serum levels [33,34].

### 5.7. Therapy of Complications

Therapy concerning specific complications like the management of necrosis and its infection haemorrhage, acute renal failure, diabetes, etc., is managed in critical care units following international guidelines not different from the general population [3].

## 6. Effect of AP on the Foetus

In pregnancy, AP has been associated with preterm labour, prematurity, and death. A severe AP may be complicated by the disruption of the function of the placenta with the death of the child. On the other hand, radiation, drugs, and anaesthesia may be potentially harmful to the baby. So, early diagnosis and correct therapy are the main goals in managing AP in pregnancy [31].

In a recent systematic review [41], normal foetal delivery was demonstrated in 42.5–100% of AP cases during pregnancy. Preterm delivery rates were between 0 and 38.1%. Total foetal loss rates were between 0 and 23.1%, foetal dismissions were between 0 and 21.2%, induced abortions were between 0 and 15.4%, and adverse foetal outcomes were between 0 and 57.41% [41].

## 7. Prevention of AP during and after Pregnancy

Most cases of AP post-partum and during pregnancy are due to biliary sludge and stones [10], and what the best therapeutic approach is to prevent new AP episodes is not defined.

Non-pregnant patients after gallstones AP must be cholecystectomised to prevent new episodes of AP [64].

During pregnancy, obesity is a risk factor for new biliary sludge and new stones. The first suggestion is to be careful with one’s diet and weight to prevent the development of new sludge and stones [10].

After biliary AP, laparoscopic cholecystectomy is safely performed during pregnancy to prevent the relapse of AP. Most of the interventions in the literature are described in the second trimester and in tertiary specialized hospitals but are not well accepted by most pregnant and new mothers, considering that small stones may spontaneously disappear. Ursodeoxycholic acid therapy is effective in dissolving small stones, and it is considered safe in the last two trimesters of pregnancy [9]; so, patients may be treated until delivery and then they must be operated on later.

Serum triglycerides increase during pregnancy and may cause AP, so all pregnant women must be screened for it; their diet must be modified, and omega-3 fatty acids may be safely added to a hypo-caloric diet when oral alimentation is not enough to keep lower triglyceride levels.

No level of alcohol has been demonstrated to be safe in pregnancy, so we discourage alcohol use during all stages of pregnancy.

In the first 2 years after delivery, AP is more frequent and biliary sludge and stones are the most frequent aetiologies. Breastfeeding, for at least 3 months, may prevent AP mainly in the youngest new mothers (less than 30 years old). After AP, in post-partum, patients must be cholecystectomised. Only when women refuse surgery may ursodeoxycholic acid be used, with ultrasound control of stone dissolution. After dissolution, ursodeoxycholic acid may be stopped but patients must be followed up by ultrasound to control stones relapse.

## 8. Potential Limitations of the Study

Several recommendations are mostly based on expert researchers’ opinions and not based on double-blind randomized controlled trials. It is difficult to perform such studies in rare diseases and in critically ill pregnant patients. Furthermore, the difficulties in managing new drugs during pregnancy limit the therapies commonly used in the general population.

## 9. Conclusions

AP in pregnancy is a challenging clinical problem to care for, with an expanding evidence-based approach. Among the different aetiological factors for AP in pregnancy, gallstones are the most common. An abdominal ultrasound is the first imaging used, but MRI and EUS may be used in pregnancy. ERCP must be avoided whenever possible as a diagnostic tool, but it can be used for therapeutic reasons. Laparoscopic surgery may be better than open surgery and can be used during pregnancy, preferably in the second trimester.

All these aggressive approaches in the diagnosis and therapy of pregnant women must be performed only in experienced tertiary institutions in light of high benefit/cost ratios.

New therapies for severe AP as well as better knowledge of pathophysiology and natural history are needed to reduce maternal and foetus mortality.

In the prevention of AP during pregnancy and the post-partum period, we recommend a diet to prevent obesity, alcohol abstinence, and baby breastfeeding for at least 3 months, especially for women less than 30 years old. We also recommend checking triglyceride levels during pregnancy and treating them appropriately. Performing ultrasounds in high-risk cases and performing cholecystectomies when clinically indicated might also be useful.

## Figures and Tables

**Table 1:** Modified Marshall scoring system for organ dysfunction.

Organ System	Score				
	0	1	2	3	4
Lung (PaO_2_/FiO_2_)	More than 400	301–400	201–300	101–	
Kidney					
(Serum creatinine, micromole/L)	Less than 135	134–169	170–310	311–439	>439
(Serum creatinine, mg/dL)	<1.4	1.4–18	1.9–3.6	3.6–4.9	>4.9
Cardiac (systolic blood pressure, mmHg)	More than 90	<90, fluid responsive	<90, not fluid responsive	<90, pH < 7.3	<90, pH < 7.2

A score of 2 or more in any system defines the presence of organ failure. Adapted from [3].

**Table 2:** Ranson’s criteria (1 point each).

On Admission		Within 48 h of Admission	
White blood cell count	>16.000 mm^3^	Haematocrit	Decreased ≥ 10 percent
Age	>55 years	BUN * increase	Increased more than 5 mg/dL
Serum glucose	More than 200 mg/dL (mmol)	Serum calcium	<8 mg/dL (2 mmol/L)
LDH **	More than 350 U/L	Arterial pO2	Less than 60 mmHg
AST ***	>280 U/L	Base deficit	>4 mEq/L
		Fluid sequestration	>6000 mL

* Blood Urea Nitrogen, ** lactate dehydrogenase, *** aspartate aminotransferase. A score of 3 points: mild pancreatitis. A score > 4 points: significant increase in the mortality rate. Adapted from [3].

**Table 3:** BISAP * score.

BUN **	>8.9 mmol/L (>25 mg/dL)	1 Point
Impaired mental status	Glasgow coma score < less than 15	1 point
SIRS ^	Presence of SIRS ^	1 point
Age	Age > 60 years	1 point
Pleural effusion	Presence	1 point

* Bedside Index of Severity in Acute Pancreatitis, ** Blood Urea Nitrogen ^ Systemic inflammatory response syndrome. A score ≥ 3 points indicates an increased risk of death (>15 percent). Adapted from [3].

**Table 4:** U.S. Food and Drug Administration categories for drugs used in pregnancy.

Category	Description
A	Well-controlled studies in pregnant women have not demonstrated an increased risk of foetal abnormalities.
B	Animal studies have not demonstrated any harm to the foetus; there are no well-controlled studies in pregnancy; Animal studies have demonstrated an adverse effect, but well-controlled studies in pregnancy have failed to show a risk to the foetus.
C	Animal studies have demonstrated an adverse effect, and there are no well-controlled studies in pregnancy, or no animal studies have been carried out, and there are no well-controlled studies in pregnancy.
D	Adequate, well-controlled, or observational studies in pregnant women have demonstrated a risk to the foetus. However, the benefits of therapy may outweigh the potential risks.
X	Well-controlled or observational studies in animals or pregnant women have shown positive evidence of foetal abnormalities. The product is contraindicated in pregnant women.

**Table 5:** Safe antibiotics during pregnancy.

Safe	Avoid	Not in First Trimester	Not in Third Trimester
Penicillins	Tetracyclines	Metronidazole	Sulfonamides
Cephalosporins	Streptomycin		Nitrofurantoin
Clindamycin	Quinolones		
Erythromycin (except estolate)			Agai in the text after table

**Table 6:** Principles guiding endoscopy in pregnancy.

Number	General Principles
1	Before endoscopy, obstetric consultation regardless of foetal gestational age.
2	Strong indication, especially in high-risk pregnancies.
3	Postpone endoscopy to 2nd trimester whenever possible.
4	Lowest effective dose of sedative drugs.
5	Only category B drugs whenever possible.
6	Reduce procedure time.
7	Patient in left lateral position or left pelvic tilt to avoid aortic or vena cava compression.
8	Individualize monitoring of foetal heart rate according to the foetus’s gestational age and available resources.
9	In the first 24 weeks of foetal gestation, you only need to confirm the foetal heart rate by Doppler before sedation and after the endoscopic procedure.
10	After 24 weeks of foetal gestation, main foetal and maternal vital parameters must be followed up
11	Absolute contraindication in ruptured membranes, placental abruption, uncontrolled eclampsia, or imminent delivery.

**Table 7:** Safety of drugs most frequently used for endoscopy during pregnancy.

Category	Drugs
A	No category A drugs used for endoscopy
B	Meperidine Naloxone Propofol Glucagon Topical anaesthetics (lidocaine)
C	Morphine Fentanyl Flumazenil
D	Benzodiazepines

## Data Availability

Not applicable.
